# Deciphering Estrus Expression in Gilts: The Role of Alternative Polyadenylation and LincRNAs in Reproductive Transcriptomics

**DOI:** 10.3390/ani14050791

**Published:** 2024-03-04

**Authors:** Mingzheng Liu, Jiahao Chen, Chunlei Zhang, Shuhan Liu, Xiaohuan Chao, Huan Yang, Asim Muhammad, Bo Zhou, Weiping Ao, Allan P. Schinckel

**Affiliations:** 1 College of Animal Science and Technology, Nanjing Agricultural University, Nanjing 210095, China; liumingzheng@stu.njau.edu.cn (M.L.); chenjiahao@stu.njau.edu.cn (J.C.); 2020105039@stu.njau.edu.cn (C.Z.); 2022105002@stu.njau.edu.cn (S.L.); 2021205020@stu.njau.edu.cn (X.C.); 2021105038@stu.njau.edu.cn (H.Y.); 2022105147@stu.njau.edu.cn (A.M.); 2 College of Animal Science and Technology, Tarim University, Alar 843300, China; 3 Department of Animal Sciences, Purdue University, West Lafayette, IN 47907-2054, USA; aschinck@purdue.edu

**Keywords:** alternative polyadenylation, estrus, LncRNA, pigs

## Abstract

**Simple Summary:**

During estrus in female pigs, a series of signs are exhibited, such as reddening and swelling of the vulva, as well as mucus discharge from the vulva, which signal ovulation and readiness for mating. We analyzed RNA-seq data from the vulva and vagina tissues of two pig breeds at different stages of estrus to elucidate their regulatory mechanisms by analyzing the differential expression genes (DEG) and long intergenic non-coding RNAs (lincRNA), as well as the utilization of alternative polyadenylation (APA) sites. The functions of these factors contribute to a better understanding of the regulatory mechanisms underlying estrus expression in gilts.

**Abstract:**

The fertility rate and litter size of female pigs are critically affected by the expression of estrus. The objective of this study was to elucidate the regulatory mechanisms of estrus expression by analyzing the differential expression of genes and long intergenic non-coding RNAs (lincRNA), as well as the utilization of alternative polyadenylation (APA) sites, in the vulva and vagina during the estrus and diestrus stages of Large White and indigenous Chinese Mi gilts. Our study revealed that the number of differentially expressed genes (DEG) in the vulva was less than that in the vagina, and the DEGs in the vulva were enriched in pathways such as “neural” pathways and steroid hormone responses, including the “Calcium signaling pathway” and “Oxytocin signaling pathway”. The DEGs in the vagina were enriched in the “Metabolic pathways” and “VEGF signaling pathway”. Furthermore, 27 and 21 differentially expressed lincRNAs (DEL), whose target genes were enriched in the “Endocrine resistance” pathway, were identified in the vulva and vagina, respectively. Additionally, we observed that 63 and 618 transcripts of the 3′-untranslated region (3′-UTR) were lengthened during estrus in the vulva and vagina, respectively. Interestingly, the genes undergoing APA events in the vulva exhibited species-specific enrichment in neural or steroid-related pathways, whereas those in the vagina were enriched in apoptosis or autophagy-related pathways. Further bioinformatic analysis of these lengthened 3′-UTRs revealed the presence of multiple miRNAs binding sites and cytoplasmic polyadenylation element (CPE) regulatory aspects. In particular, we identified more than 10 CPEs in the validated lengthened 3′-UTRs of the *NFIX*, *PCNX4*, *CEP162* and *ABHD2* genes using RT-qPCR. These findings demonstrated the involvement of APA and lincRNAs in the regulation of estrus expression in female pigs, providing new insights into the molecular mechanisms underlying estrus expression in pigs.

## 1. Introduction

The significance of estrus expression in sows during estrus is critical for pig reproduction [1,2]. Prolonged and apparent estrus expression is vital for timely insemination, while concealed or less apparent estrus expression leads a lower conception rate [3]. Additionally, the absence of estrus expression results in the culling of a significant number of gilts from the breeding herd, posing one of the major unresolved challenges in the pig farming industry [4]. During estrus, female pigs exhibit various signs, including vaginal mucus secretion, vulva reddening, and swelling, indicating ovulation and readiness for mating [5,6]. While previous studies have acknowledged these signs, and our recent research corroborates them [7,8,9], there remains a gap in understanding how estrus expression regulation changes throughout the estrus cycle.

In eukaryotes, there is a specific “CA” dinucleotide site at the 3′-untranslated regions (3′-UTRs) of pre-mRNA, known as the polyadenylation signal site (PAS), where cleavage occurs and approximately 100–200 adenosine acid nucleotides are added to a poly (A) tail to complete polyadenylation [10]. Genome-wide studies have shown that 60–70% of human genes have multiple polyadenylation sites [11,12]. Some polyadenylation sites have also been identified in pigs [13,14]. Alternative polyadenylation (APA) refers to the differential use of multiple PAS within a given pre-mRNA [15]. The use of alternative polyadenylation sites is an important mechanism for generating different isoforms of 3′-UTRs that regulate gene expression levels, mRNA stability, localization, and translation after transcription [16,17,18,19]. Different lengths of 3′-UTRs produce variable miRNA binding sites, with lengthened 3′-UTRs increasing the number of miRNA binding sites, thereby enhancing mRNA transcription inhibition [20]. The 3′-UTRs contain important elements, such as hexameric PAS and cytoplasmic polyadenylation elements (CPE), which regulate cytoplasmic polyadenylation and interact with post-transcriptional factors to influence mRNA degradation and translation [21,22]. APA regulates gene expression and function in a cell- and tissue-specific manner [10,23], and it has been demonstrated that long non-coding RNAs (lncRNAs) exert impacts pig traits [24], especially those related to pig reproductive traits [25]. 

The exploration into long non-coding RNAs (lncRNAs) has unveiled their significant impact on pig traits, particularly those linked to reproductive functions. Within this realm, long intergenic non-coding RNAs (lincRNAs), which do not intersect with annotated coding sequences, stand out for their unique functions and regulatory mechanisms [26]. They account for a significant portion of the lncRNA population in humans [27], highlighting their potential impact on gene regulation. Building on this foundation, we hypothesize that lincRNA and APA may be involved in the regulation of estrous performance, and there is a potentially groundbreaking interaction between lincRNAs and APA mechanisms within the vaginal and vulva tissues of female pigs. This interaction could play a pivotal role in regulating estrus expression, suggesting a layered and sophisticated regulatory network. The potential for lincRNAs to influence the selection of APA sites, and thereby the generation of mRNA isoforms with varying 3′-UTR lengths, introduces a complex dynamic in gene expression regulation [28]. This complexity is further magnified by the capacity of APA to alter the landscape of miRNA binding sites on mRNAs, which could be modulated by lincRNAs either directly or indirectly. However, this regulatory mechanism is complex, and lincRNA may potentially participate in the interaction of APA through miRNA or other mediators [29]. Such interactions may influence not only the stability and localization of mRNAs but also their translational efficiency [30], offering a rich tapestry of regulatory possibilities that could affect reproductive traits and beyond.

Our previous studies have demonstrated differences in the color of the vulva and vaginal mucus between the estrus and diestrus stages. We have conducted a gene set enrichment analysis (GSEA) to analyze the functions of genes in the vulva and vaginal tissues [31]. In the present study, vaginal and vulva tissues from European Large White gilts and indigenous Chinese Mi gilts were utilized to analyze mRNA, APA, and lincRNAs during different stages of estrus cycles. The expression characteristics and potential functions of these APA and lincRNAs sites provide new insights into the regulation of estrus expression in female pigs. 

## 2. Materials and Methods

This study was approved by the Animal Care and Use Committee of Nanjing Agricultural University (SYXK2017-0027).

### 2.1. Sample Collection

This study involved 60 gilts (30 Large White and 30 Mi) housed at Yong Kang Agricultural Science and Technology Co., Ltd., Changzhou, Jiangsu Province, China. Selected before their second estrus cycle, the gilts were raised under standardized conditions with free access to water and a standardized feeding regimen. Estrus detection was performed twice daily using visual assessments and the back-pressure test for standing reflex, alongside daily boar introduction for stimulation. Gilts exhibiting strong estrus expression were selected for sample collection during their second and third estrus cycles. Selected gilts were humanely slaughtered on the first and tenth days of the third estrus cycle for vulva and vaginal sample collection, as described previously [32]. 

The samples were collected during the estrus and diestrus stages. For the estrus stage, collection occurred on the first day a gilt exhibited the standing reflex. For the diestrus stage, samples were taken on the 10th day after the third estrus cycle. Three Large White and three Mi gilts were humanely euthanized at each stage (designated as LE and ME for estrus, and LD and MD for diestrus, respectively). Careful dissection of the vagina and vulva tissues was performed on 6 Large White and 6 Mi pigs, from which samples were collected and stored at −80 °C for subsequent RNA extraction.

### 2.2. Sequencing of Samples

Trizol reagent (Invitrogen, Carlsbad, CA, USA) was used to extract total RNA from 24 samples. Agarose gel electrophoresis was performed to evaluate the samples for evidence of degradation and contamination. RNA concentration and purity were assessed using a NanoPhotometer^®^ spectrophotometer (IMPLEN, Los Angeles, CA, USA). The samples were sequenced using a DNBSEQ platform (DNBSEQ technology, Shenzhen, China). Sequencing was performed as described previously for library construction methods and sequencing procedures [31].

### 2.3. LincRNAs Identification 

Twelve vagina and 12 vulva RNA-seq libraries were constructed separately. To obtain clean reads, the tools Trimmomatic [33] and SOAPnuke [34] were used for pre-processing. Then, the quality of the clean reads was evaluated using Q20, Q30, and GC content metrics. Only high-quality clean reads were selected for subsequent analysis. The selected clean reads were aligned against the pig reference genome (Sscrofa11.1, version Sus_scrofa.Sscrofa11.1.107) using HISAT2 aligner [35], and the bam files were obtained using Samtools [36] and employed for further analysis. 

To identify the lincRNAs, we implemented the following steps: (1) We extracted transcripts classified as ‘U’ from the reference genome. (2) Only transcripts with more than two exons and a minimum length of 200 base pairs were retained. (3) We employed NCBI NR, UniRef 90, CPC2, and Pfam to filter out transcripts with coding potential. (4) Transcripts expressed in at least one sample were selected as the final lincRNA candidates. To explore the cis-regulation of lincRNAs, protein-coding genes located within 100 kb upstream and downstream of the lincRNA were identified using BEDTools [37].

### 2.4. Analysis of Differential Expression and Function Enrichment Analysis

We aligned protein-coding genes and identified long intergenic non-coding RNAs (lincRNAs) against the pig genome reference sequence (Sscrofa11.1, version Sus_scrofa.Sscrofa11.1.107) to perform differential expression analysis. For quantifying reads across each sample, we utilized the “featureCounts” software (Version 2.0.1). Differential expression was analyzed using the “DESeq2” package, applying significance thresholds of an adjusted *p*-value < 0.05 and an absolute log2(fold change) > 1. This approach enabled the identification of transcripts that were differentially expressed across all comparison groups in the two tissues (LE vs. ME, LD vs. LE, LD vs. MD, and MD vs. ME). Gene Ontology (GO) enrichment and Kyoto Encyclopedia of Genes and Genomes (KEGG) pathway analyses of the genes were performed by the KOBAS database (http://kobas.cbi.pku.edu.cn/, accessed on 25 November 2023) [38]. A *p*-value threshold of less than 0.05 was considered statistically in regard to enrichment terms or pathways.

### 2.5. Analysis of Alternative Polyadenylation in the 3′-UTR

DaPars (https://github.com/ZhengXia/dapars, accessed on 26 December 2023) was used to analyze the use of APA sites in the vagina and vulva during the estrus and diestrus stages. First, the bam files were converted to wig files, and then the wig files of different groups were compared to obtain transcript subtypes with different 3′-UTR lengths. By calculating the percentage change in the usage index of distal poly(A) sites (PDUI), the usage of proximal and distal poly(A) sites was quantified. Finally, ΔPDUI (defined as the average PDUI of one group − the average PDUI of another group) was calculated to indicate the shortened or lengthened 3′-UTR length in the sample. An adjusted *p*-value less than 0.05 and |ΔPDUI| greater than 0.05 was considered statistically for the APA site [22]. 

### 2.6. Analysis of 3′-UTR Sequences and Functional Elements

The IGV tool was used to analyze and visualize the length and abundance information of the 3′-UTR in each sample [39]. First, the reference genome (Sscrofa11.1, version Sus_scrofa.Sscrofa11.1.107) and annotation files were imported into IGV, followed by the import of bam files for each sample. Subsequently, the lengths and differences of the identified 3′-UTRs were examined. Then, we used an online program (http://genome.crg.es/CPE/server.html, accessed on 5 November 2023) to predict the types, numbers, and positions of CPE sites (CPENC, CPEC, HEXA and PBE) [40]. Miranda was used to analyze the binding sites of miRNAs in the extended sequences of 3′-UTRs [41], and the predicted results were visualized using Cytoscape (https://cytoscape.org, accessed on 2 December 2023).

### 2.7. RT-qPCR Verification

Total cDNA was synthesized using a reverse transcriptase kit and subjected to RT-qPCR using a SYBR Green Master Mix (Vazyme Biotech, Nanjing, China). The PCR reactions were performed in triplicate, and primers of the lengthened 3′-UTRs were designed using the Primer Premier 5 software (Table S1). The relative gene expression was determined using the 2^−∆∆Ct^ method and normalized to the reference gene *GAPDH*. Expression data analysis was carried out using GraphPad Prism (version 8.0, San Diego, CA, USA). The t-test for independent samples was used to analyze statistical significance and significant differences at *p*-value < 0.05. All reported results were presented as means ± standard error of the mean (SEM).

## 3. Results

### 3.1. Analysis of Differentially Expressed Genes in the Vulva and Vagina

**Figure 1:**
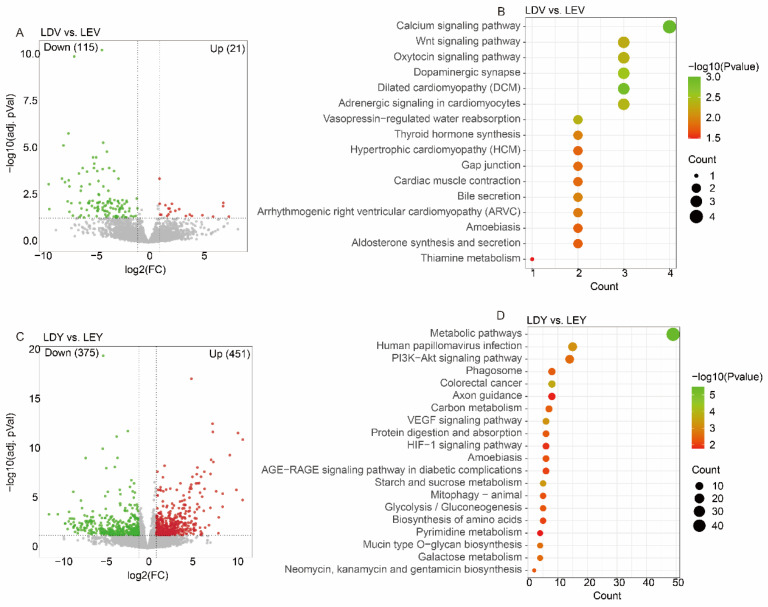
Analysis of differentially expressed genes in Large White gilts. (**A**) A volcano plot displays differentially expressed genes in the LDV (vulva samples of Large White gilts at Diestrus stage) vs. LEV (vulva samples of Large White gilts at Estrus stage) comparison, with significance thresholds set at FPKM > 1, adjusted *p*-value < 0.05 and |log2 (fold change)| > 1. (**B**) KEGG enrichment analysis of differentially expressed genes in the LDV vs. LEV comparison. (**C**) A volcano plot displays differentially expressed genes in the LDY (vagina samples of Large White gilts at Diestrus stage) vs. LEY (vagina samples of Large White gilts at Estrus stage) comparison, with significance thresholds set at FPKM > 1, adjusted *p*-value < 0.05 and |log2 (fold change)| > 1. (**D**) KEGG enrichment analysis of differentially expressed genes in the LDY vs. LEY comparison.

With optimal sequencing and alignment quality (Table S2), we identified 136 differentially expressed genes (DEGs) in the LDV (vulva samples of Large White gilts at Diestrus stage) vs. LEV (vulva samples of Large White gilts at Estrus stage) comparison, including 21 upregulated genes and 115 downregulated genes (Figure 1A). Similarly, we have observed 826 DEGs in the LDY (vagina samples of Large White gilts at Diestrus stage) vs. LEY (vagina samples of Large White gilts at Estrus stage) comparison, with 451 genes upregulated and 375 genes downregulated (Figure 1C). Notably, these DEGs in the vulva were significantly enriched in pathways such as the “Calcium signaling pathway”, “Wnt signaling pathway” and “Oxytocin signaling pathway” (Figure 1B). These DEGs in the vagina were significantly enriched in pathways such as “Metabolic pathways”, “PI3K—kt signaling pathway” and “VEGF signaling pathway” (Figure 1D).

In Chinese Mi gilts, we detected 53 DEGs in the MDV (Vulva samples of Mi gilts at Diestrus stage) vs. MEV (Vulva samples of Mi gilts at Estrus stage) comparison (Figure S1A), which were significantly enriched in pathways like the “Wnt signaling pathway” and “Toll—like receptor signaling pathway” (Figure S1B). Simultaneously, we also discovered 3528 DEGs in the MDY (vagina samples of Mi gilts at Diestrus stage) vs. MEY (vagina samples of Mi gilts at Estrus stage) comparison (Figure S1C), similarly showing significant enrichment in pathways such as “Metabolic pathways”, “Wnt signaling pathway” and “GnRH signaling pathway” (Figure S1D). The DEGs of all comparison groups are shown in Table S3.

### 3.2. Analysis of Differentially Expressed lincRNAs in the Vulva and Vagina

**Figure 2:**
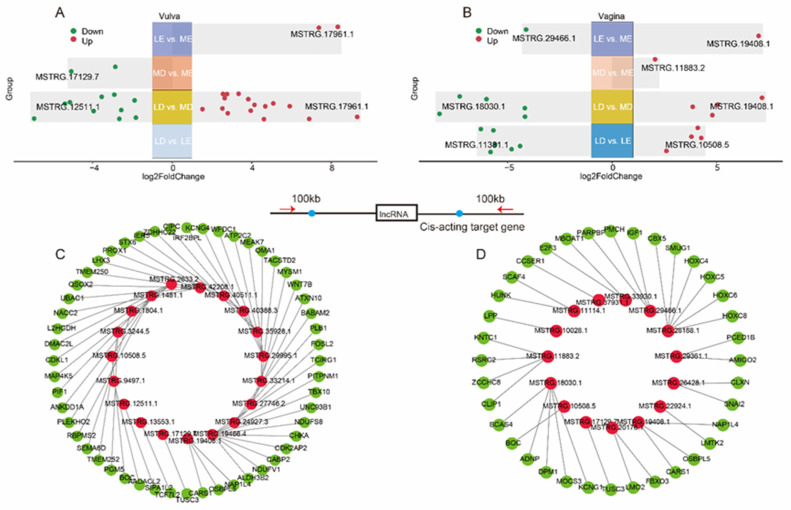
Analysis of differentially expressed lincRNAs (**A**). The distribution of differentially expressed lincRNAs in four comparisons of the vagina (**B**). Cis-acting target genes of lincRNA in the vulva (**C**), with red indicating lincRNA and green indicating target genes. Cis-acting target genes of lincRNA in the vulva (**D**).

To fully explore the lincRNAs that are associated with estrus expression in gilts, we have divided the sequencing samples into two cross-stage comparisons (LD vs. LE, MD vs. ME) and two cross-breed comparisons (LE vs. ME, LD vs. MD). Specifically, there were 0, 26, 2 and 2 differentially expressed lincRNAs (DELs) detected in LD vs. LE, LD vs. MD and MD vs. ME and LE vs. ME comparisons of the vulva, respectively (Figure 2A). Moreover, there were 10, 9, 1 and 2 DELs detected in LD vs. LE, LD vs. MD and MD vs. ME and LE vs. ME comparisons of the vagina, respectively (Figure 2B). The DELs of all comparison groups are shown in Table S4.

Among the four comparisons of the vulva, a total of 27 DELs were identified (Figure 2A), and 53 potential cis-acting target genes were predicted using cis-acting analysis (Figure 2C, Table S5). These target genes were found to be significantly enriched in pathways such as “Metabolic pathways”, “Oxidative phosphorylation” and “Hippo signaling pathway” (Figure S2A). Among the four comparisons of the vagina, a total of 21 DELs were identified (Figure 2B), and 36 potential cis-acting target genes of these lincRNAs were predicted (Figure 2D, Table S5). These target genes were found to be significantly enriched in pathways such as “N—Glycan biosynthesis”, “Endocrine resistance” and “mTOR signaling pathway” (Figure S2B).

### 3.3. Identification and Analysis of APA Sites in the Estrus Cycles

**Figure 3:**
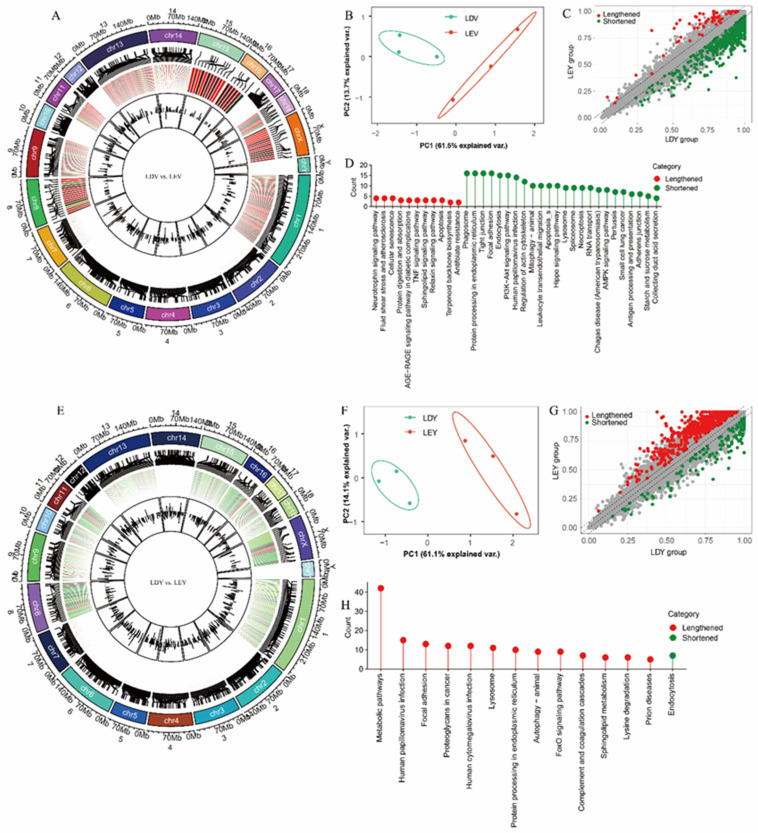
Identification and analysis of APA sites in the estrus cycles of Large White gilts. The distribution of APA sites in the vulva (**A**) and vagina (**E**). PCA of transcripts undergoing APA vulva (**B**) and vagina (**F**). Distribution of shortened and lengthened 3′-UTRs vulva (**C**) and vagina (**G**). Functional enrichment analysis of transcripts undergoing APA vulva (**D**) and vagina (**H**). LDV (vulva samples of Large White gilts at Diestrus stage), LEV (vulva samples of Large White gilts at Estrus stage), LDY (vagina samples of Large White gilts at Diestrus stage), LEY (vagina samples of Large White gilts at Estrus stage).

During the estrus and diestrus stages in Large White gilts, we found changes in the 3′-UTR length of 631 (Figure 3A) and 748 (Figure 3E) transcripts in the vulva and vagina, respectively. Principal component analysis (PCA) revealed separate clustering of samples from the vulva (Figure 3B) or vagina (Figure 3F) tissues at different estrus stages. Specifically, in the genes undergoing at the APA site in the vulva, there were 568 transcripts with shortened 3′-UTRs during estrus and 63 transcripts with lengthened 3′-UTRs (Figure 3C). Genes with lengthened 3′-UTRs during estrus were enriched in 11 pathways, including some interesting pathways such as the “Neurotrophin signaling pathway”, “TNF signaling pathway”, and “Apoptosis”, while genes with shortened 3′-UTRs were enriched in 24 pathways, primarily pathways like “Mitophagy-animal”, “Spliceosome”, and “Adherens junction” (Figure 3D, Table S6). In the vagina, only 130 transcripts have shortened 3′-UTRs, while 618 transcripts have lengthened 3′-UTRs (Figure 3G). Genes with lengthened 3′-UTRs during estrus were enriched in 13 pathways, including the pathways “Metabolic pathways”, “Autophagy-animal”, and “FoxO signaling pathway”, while genes with shortened 3′-UTRs were only enriched in the “Endocytosis” pathway (Figure 3H, Table S7). 

In Mi gilts, we observed changes in the 3′-UTR length of 663 (Figure S3A) and 353 (Figure S3E) transcripts during the estrus cycle in the vulva and vagina, respectively. There are 179 (Figure S3C) and 151 (Figure S3G) transcripts with shortened 3′-UTRs in the vulva and vagina, respectively. Interestingly, the “Steroid biosynthesis” and “Cell adhesion molecules (CAMs)” pathways were enriched in the vulva (Figure S3D, Table S6); whereas the “HIF-1 signaling pathway” was enriched in the vagina (Figure S3H, Table S7).

Furthermore, we also conducted an analysis of cross-tissue APA events in these two pig breeds. while 2628 (Figure S4A) transcripts had different 3′-UTR lengths in the vulva vs. vagina in Large White gilts. Compared to the vagina, genes with lengthened 3′-UTRs in the vulva were enriched in 20 pathways, including the “Metabolic pathways”, “PI3K-Akt signaling pathway”, and “Spliceosome”, while genes with shortened 3′-UTRs were enriched in “Glycerophospholipid metabolism” and “Ether lipid metabolism” pathways (Figure S4D). In Mi gilts, a total of 1902 (Figure S3A) transcripts with different 3′-UTR lengths were identified in the vulva vs. vagina (Figure S4D). Compared to the vagina, the vulva of Mi gilts had 133 transcripts with shortened 3′-UTRs and 1769 with lengthened 3′-UTRs (Figure S3G). In addition, genes with 3′-UTR alterations in both the vulva and vagina also exhibited specific enrichment in the “Oocyte meiosis” pathway (Figure S3H, Table S8). 

### 3.4. Visualization and Validation of 3′-UTR Expression Abundance

**Figure 4:**
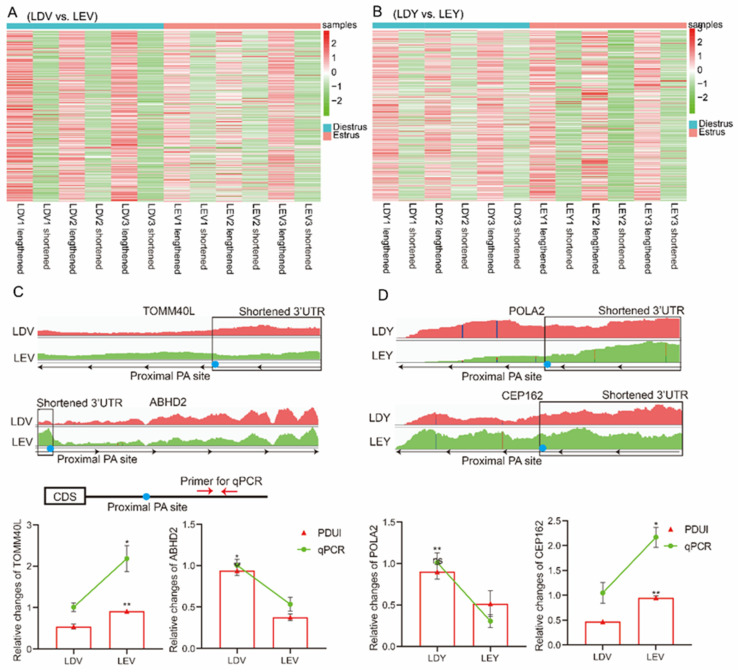
Visualization and validation of 3′-UTR expression abundance of Large White gilts. The heatmap depicts the expression patterns of lengthened and shortened 3′-UTRs in the vulva (**A**) and vagina (**B**) during the estrus and diestrus stages. IGV used to show 3′-UTR lengths and abundance of *TOMM40L*, *ABHD2* (**C**), *POLA2*, *CEP162* (**D**) genes. RT-qPCR was employed to validate the expression levels of their lengthened 3′-UTRs. * *p* < 0.05; ** *p* < 0.01.

To investigate the role of APA sites in the 3′-UTR, we characterized the expression abundance of the 3′-UTR in each sample. In the vulva, the heatmap revealed that the expression abundance of lengthened 3′-UTRs was lower during the diestrus stage and higher during the estrus stage, whereas the expression of shortened 3′-UTRs was higher during the diestrus stage and lower during the estrus stage (Figure 4A). In the vagina, the heatmap showed that the expression abundance of lengthened 3′-UTRs was higher during the estrus stage compared to the diestrus stage, while the expression of shortened 3′-UTRs was lower during the estrus stage (Figure 4B). Specifically, the expression of the *TOMM40L* gene shortened 3′-UTR was higher in the vulva during the diestrus stage compared to the estrus stage, while the expression of *ABHD2* gene shortened 3′-UTR was higher in the vulva during the estrus stage (Figure 4C). Moreover, the expression of the *POLA2* gene shortened 3′-UTR was higher in the vagina during the diestrus stage compared to the estrus stage, while the expression of the *CEP162* gene shortened 3′-UTR was higher in the vagina during the estrus stage (Figure 4D). We designed primers specific to each gene located in the lengthened 3′-UTR and found that the relative expression results were consistent with the results obtained using IGV.

Moreover, when comparing the vulva, it was observed that the expression abundance of lengthened 3′-UTRs was higher in the vagina, whereas the expression of shortened 3′-UTRs was lower (Figure S5A). The expression of the *NFIB* gene shortened 3′-UTR was lower in the vagina compared to the vulva, while the expression of the *PCNX4* gene shortened 3-UTR was higher in the vagina (Figure S5B). 

### 3.5. Regulatory Elements in Lengthened 3′-UTR

**Figure 5:**
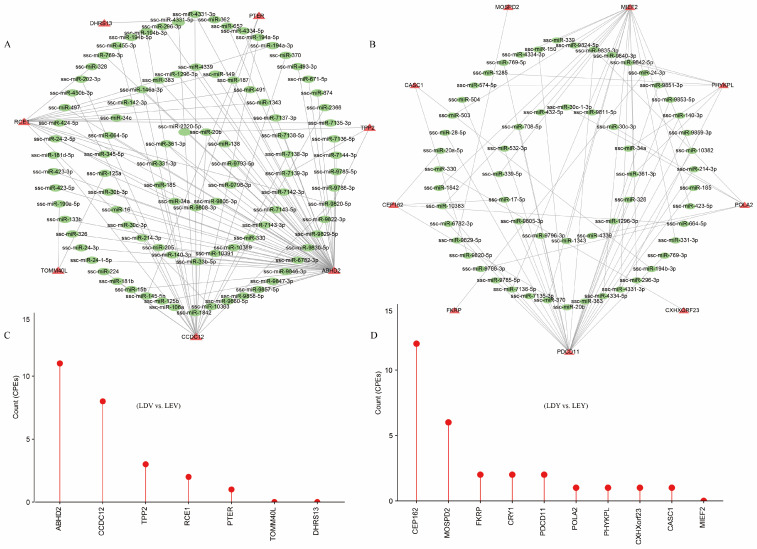
Regulatory elements in lengthened 3′-UTRs of Large White gilts. The miRNA-gene interactome network of lengthened 3′-UTRs in the vulva (**A**) and vagina (**B**) during the estrus and diestrus stages. The green color represents miRNAs, while the red color represents genes with lengthened 3′-UTRs. The CPEs on the lengthened 3′-UTRs in the vulva (**C**) and vagina (**D**) during the estrus and diestrus stages.

By filtering for significant transcripts with |ΔPDUI| ≥ 0.35, we obtained 7, 10, and 20 high-confidence transcripts in the LDV vs. LEV, LDY vs. LEY and LY vs. LV comparisons, respectively (Table S9). We analyzed the potential miRNA binding sites within these binding sequences. We found that among the seven genes in the LDV vs. LEV comparison, a total of 95 miRNA binding sites were predicted in their lengthened 3′-UTRs (Figure 5A). Among the 10 genes in the LDY vs. LEY comparison, a total of 59 miRNA binding sites were predicted in their lengthened 3′-UTRs (Figure 5B). Furthermore, a total of 114 miRNA binding sites were predicted in the lengthened 3′-UTRs of the 10 genes in the LY vs. LV comparison (Figure S5C). These miRNAs could be one of the reasons for regulating the abundance of 3′-UTRs or mRNAs expression. Furthermore, we have also conducted predictions on the CPEs present in the lengthened 3′-UTRs of these genes (Table S10). Among them, nine genes were found to harbor more than 10 CPEs, including the *NFIX*, *PCNX4*, *CEP162* and *ABHD2* genes. These findings provide important clues for further research on the transcriptional regulation of genes.

## 4. Discussion

In the present study, the number of DEGs in the vulva is much lower than that in the vagina, which may be related to the vagina having more physiological functions than the vulva. Interestingly, we identified enrichment of the “Calcium signaling pathway”, “Oxytocin signaling pathway” and “Dopaminergic synapse” pathways in DEGs in the vulva, which may be related to the vulva’s response to steroid hormones and neural regulation of estrus expression. In addition, the vagina is enriched with the “Metabolic pathways” and “VEGF signaling pathway”; this is because there are target genes of estrogen in the vagina, which may be involved in vaginal mucus secretion and metabolic functions [42]. LncRNAs have been increasingly found to play a role in the reproductive regulation of pigs [43,44]. In our earlier studies, lincRNAs in the ovaries during different estrus phases were identified [7]. To comprehensively understand the regulation mechanism of the vulva and vagina during the estrus cycle, we characterized all DELs that could potentially be involved. The results indicate that the cis-acting target genes of these lincRNAs may be associated with estrus expression. For instance, the enrichment of target genes of DELs was observed in the “Endocrine resistance” pathway in the vagina.

Recent studies have unveiled the role of APA sites, a process involving selective splice-site usage, in regulating mRNA expression, translation, and localization of protein-coding genes [45,46]. As a transcriptional modulator, APA exerts its influence by directly influencing the length of a gene’s 3′-UTR, thereby participating in the regulation of organismal homeostasis and phenotypic traits [23,47,48,49]. In this study, we employed a RNA-seq to identify numerous APA events associated with estrus expression in the vulva and vagina tissues of gilts [50]. Remarkably, these APA events were observed not only in indigenous Chinese pig breeds but also in European pig breeds, suggesting their ubiquitous presence throughout different estrus cycles and possibly serving as a crucial modulatory mechanism of estrus expression in female pigs. In this study, our findings reveal that, regardless of whether in the vagina or vulva, the estrus stage induces APA in hundreds of genes, resulting in 3′-UTR elongation or shortening. Additionally, we have identified distinct stage specificity in APA sites [51]. By comparing APA sites in the vulva and vagina, we observe a greater tissue specificity, with a much larger number of genes involved in 3′-UTR modifications compared to genes involved in different estrus stages [52]. This suggests that APA-mediated regulation of gene expression may be exquisitely precise and sensitive. 

Investigations on APA have revealed its implications in the exploration of numerous pathologies and traits [53,54]. In our current study, we have identified genes undergoing APA in the vulva of Large White gilts that are enriched in the “Neurotrophin signaling” pathway, whereas in Mi gilts, they are enriched in “Steroid synthesis” pathways. It is well-established that estrus expression in pigs is initiated by ovarian steroid hormones and further regulated through the hypothalamic–pituitary axis [55,56]. Therefore, the hypothalamic–pituitary–ovary (HPO) axis stands as the principal regulatory mechanism for the estrus cycle, while the vulva and vagina serve as carriers of estrus expression [57]. Consequently, the enrichment of genes related to neural and hormonal functions is to be expected, as these genes respond to the regulation of steroid hormones and neural activities. The 3′-UTRs of genes harbor numerous regulatory elements that are involved in mRNA translation, expression, localization, and more [58]. Through analysis of these lengthened 3′-UTRs, we have discovered multiple miRNA binding sites and CPE regulatory elements. These findings suggest that APA may be involved in the transcriptional regulation of mRNA to facilitate rapid responses in the vulva and vagina during estrus in gilts. Although we have quantitatively validated APA events for several representative genes, their involvement in the regulation of estrus expression remains unknown and represents a future direction for our research.

In discussing our findings, we must acknowledge a limitation: the small sample size, with only three gilts per group, which could impact the statistical power of our results. Future studies with larger sample sizes are essential to validate our findings and to explore the complex biology of estrus expression in pigs more thoroughly.

Taken together, we conducted a comprehensive analysis of the transcriptional differences, including protein-coding genes, lincRNAs, and APA, in the vulva and vagina of gilts at the stages of estrus and diestrus. These findings provide important insights into the transcriptional regulatory mechanisms of mRNA, as well as the involvement of lincRNAs and APA sites in the homeostasis and phenotypic traits. Further research on the functions and regulatory mechanisms of lincRNAs and APA across diverse biological processes promises to provide a more comprehensive understanding of the complexity and diversity of gene expression regulation. Our study provides new research perspectives for understanding the regulation of estrus expression in gilts.

## 5. Conclusions

In this study, we identified differentially expressed genes and lincRNA in the vulva and vagina at different stages of estrus. Differentially expressed genes during estrus in the vulva were enriched in the “Calcium signaling pathway” and “Oxytocin signaling pathway”, while those in the vagina were enriched in the “Metabolic pathways” and “VEGF signaling pathway”. The target genes of differentially expressed lincRNA were enriched in the “Endocrine resistance” pathway. Furthermore, genes undergoing APA in the vulva are enriched in neural or steroid-related pathways, while those in the vagina are enriched in apoptotic or autophagy-related pathways. Further analysis of this lengthened 3′-UTR revealed the presence of multiple miRNA binding sites and CPE regulatory elements. These findings demonstrated that lincRNAs and APA regulate functional genes involved in estrus expression in gilts in a stage-dependent manner, providing new insights into the molecular regulatory mechanisms of estrus expression in gilts.

## Data Availability

The datasets generated and/or analyzed during the current study are available from the corresponding author upon reasonable request.

## Supplementary Materials

The following supporting information can be downloaded at: https://www.mdpi.com/article/10.3390/ani14050791/s1, Figure S1: Analysis of Differentially Expressed genes of the Mi gilts; Figure S2: Functional analysis of lincRNA target genes; Figure S3: Identification and analysis of APA sites in the estrus cycles of the Mi gilts; Figure S4: Identification and analysis of cross-tissue APA events. Figure S5: Regulatory elements in lengthened 3′-UTRs of cross-tissue APA events. Table S1. Primer for the 3′-UTR of genes for real-time quantitative PCR; Table S2: Alignment of samples used for RNA sequencing; Table S3: The DEGs of all comparison groups; Table S4: The DELs of all comparison groups; Table S5: Cis-acting target genes of differentially expressed lincRNAs; Table S6: Functional enrichment analysis of genes undergoing APA in the vulva during the estrus and diestrus stages; Table S7: Functional enrichment analysis of genes undergoing APA in the vagina during the estrus and diestrus stages; Table S8: Functional enrichment analysis of genes undergoing APA in the vulva and vagina; Table S9: The transcripts with |ΔPDUI| ≥ 0.35; Table S10: Overview of the CPEs present in the lengthened 3′-UTRs of these genes with |ΔPDUI| ≥ 0.35.
