# Supplementary material for: Deciphering Estrus Expression in Gilts: The Role of Alternative Polyadenylation and LincRNAs in Reproductive Transcriptomics
Source: Animals (Basel). 2024 Mar 4;14(5):791. doi: 10.3390/ani14050791 (PMC10931002; doi:10.3390/ani14050791)
Supplement: Supplementary file 1 [file animals-14-00791-s001.zip › Supplementary Figure.pdf]

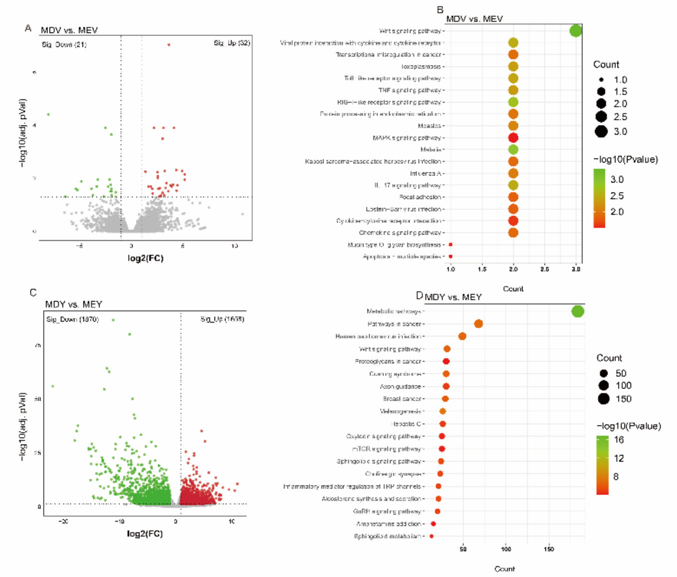

Supplementary Figure S1. Analysis of Differentially Expressed genes of the Mi gilts. The volcano plot displays differentially expressed genes in the MDV (Vulva samples of Mi gilts at Diestrus stage) vs. MEV (Vulva samples of Mi gilts at Estrus stage) comparison (A). KEGG enrichment analysis of differentially expressed genes in the vulva (B). The volcano plot displays differentially expressed genes in the MDY (Vagina samples of Mi gilts at Diestrus stage) vs. MEY (Vagina samples of Mi gilts at Estrus stage) comparison (C). KEGG enrichment analysis of differentially expressed genes in the vagina (D).

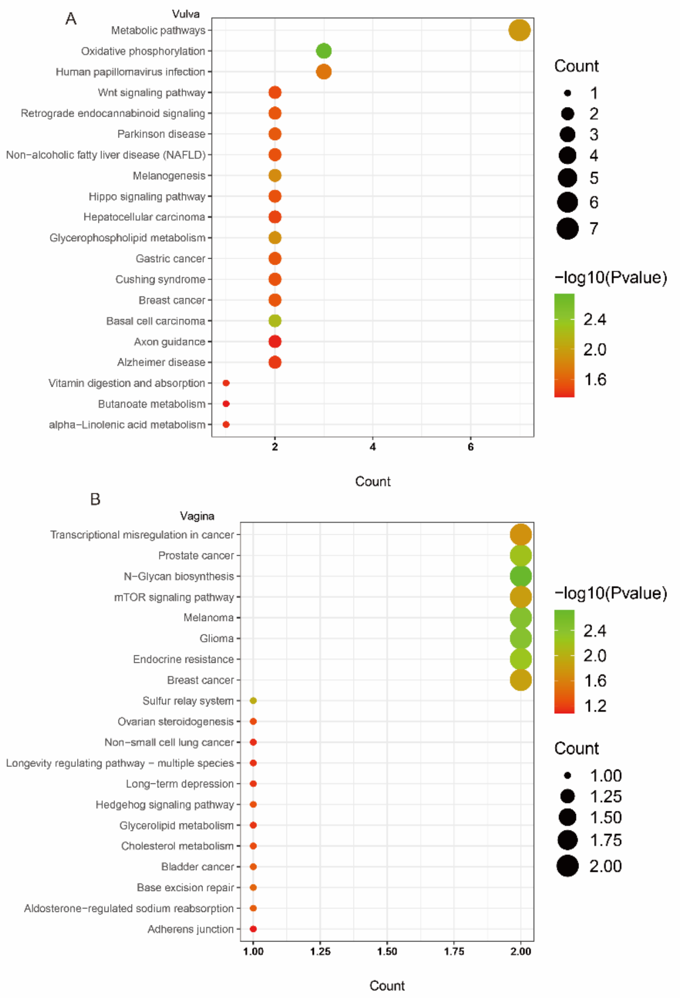

Supplementary Figure S2. Functional analysis of lincRNA target genes. KEGG enrichment analysis of lincRNA target genes in the MDV vs. MEV comparison (A). KEGG enrichment analysis of lincRNA target genes in the MDY vs. MEY comparison (B).

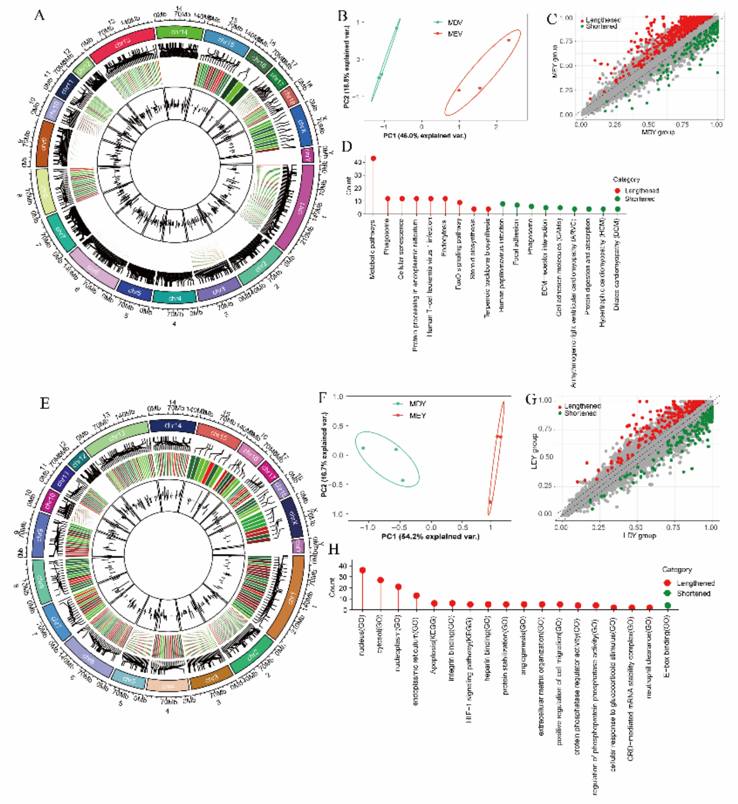

Supplementary Figure S3. Identification and analysis of APA sites in the estrus cycles of the Mi gilts. The distribution of APA sites in the vulva (A) and vagina (E). PCA of transcripts undergoing APA vulva (B) and vagina (F). Distribution of shortened and lengthened 3'-UTRs vulva (C) and vagina (G). Functional enrichment analysis of transcripts undergoing APA vulva (D) and vagina (H).

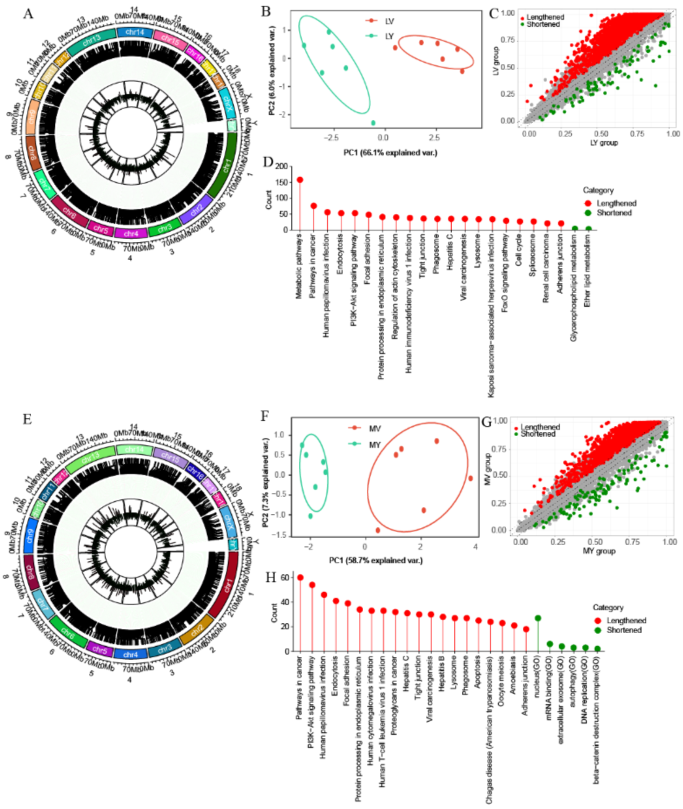

Supplementary Figure S4. Identification and analysis of cross-tissue APA events. The distribution of APA sites in the Large White gilts (A) and Mi gilts (E). PCA of transcripts undergoing APA in the Large White gilts (B) and Mi gilts (F). Distribution of shortened and lengthened 3'-UTRs in the Large White gilts (C) and Mi gilts (G). Functional enrichment analysis of transcripts undergoing APA in the Large White gilts (D) and Mi gilts (H).

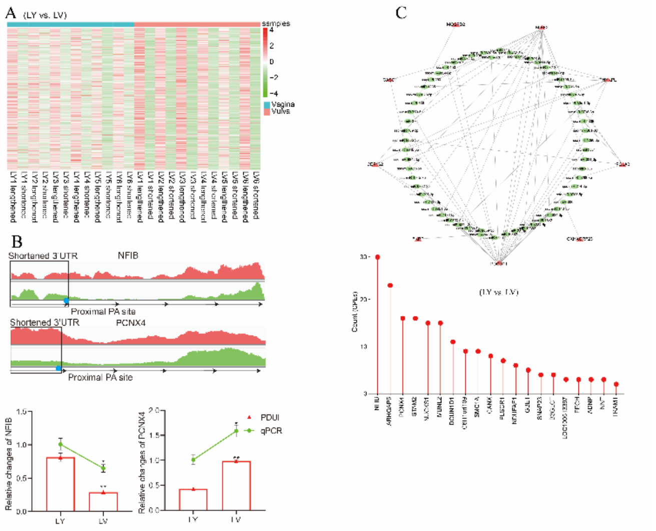

Supplementary Figure S5. Regulatory elements in lengthened 3'-UTRs of cross-tissue APA events. The heatmap depicts the expression patterns of lengthened and shortened 3'-UTRs of cross-tissue APA (A). IGV to show 3'-UTRs length and abundance of NFIB and PCNX4 (B) gene. RT-qPCR was employed to validate the expression levels of their lengthened 3'-

UTRs. The miRNA-gene interactome network of lengthened 3'-UTRs of cross-tissue APA (C). The green color represents miRNAs, while the red color represents genes with lengthened 3'-UTR. The diagram below presents the CPEs.
