# Supplementary figures and images for: Deciphering Estrus Expression in Gilts: The Role of Alternative Polyadenylation and LincRNAs in Reproductive Transcriptomics
Source: Animals (Basel). 2024 Mar 4;14(5):791. doi: 10.3390/ani14050791 (PMC10931002; doi:10.3390/ani14050791)

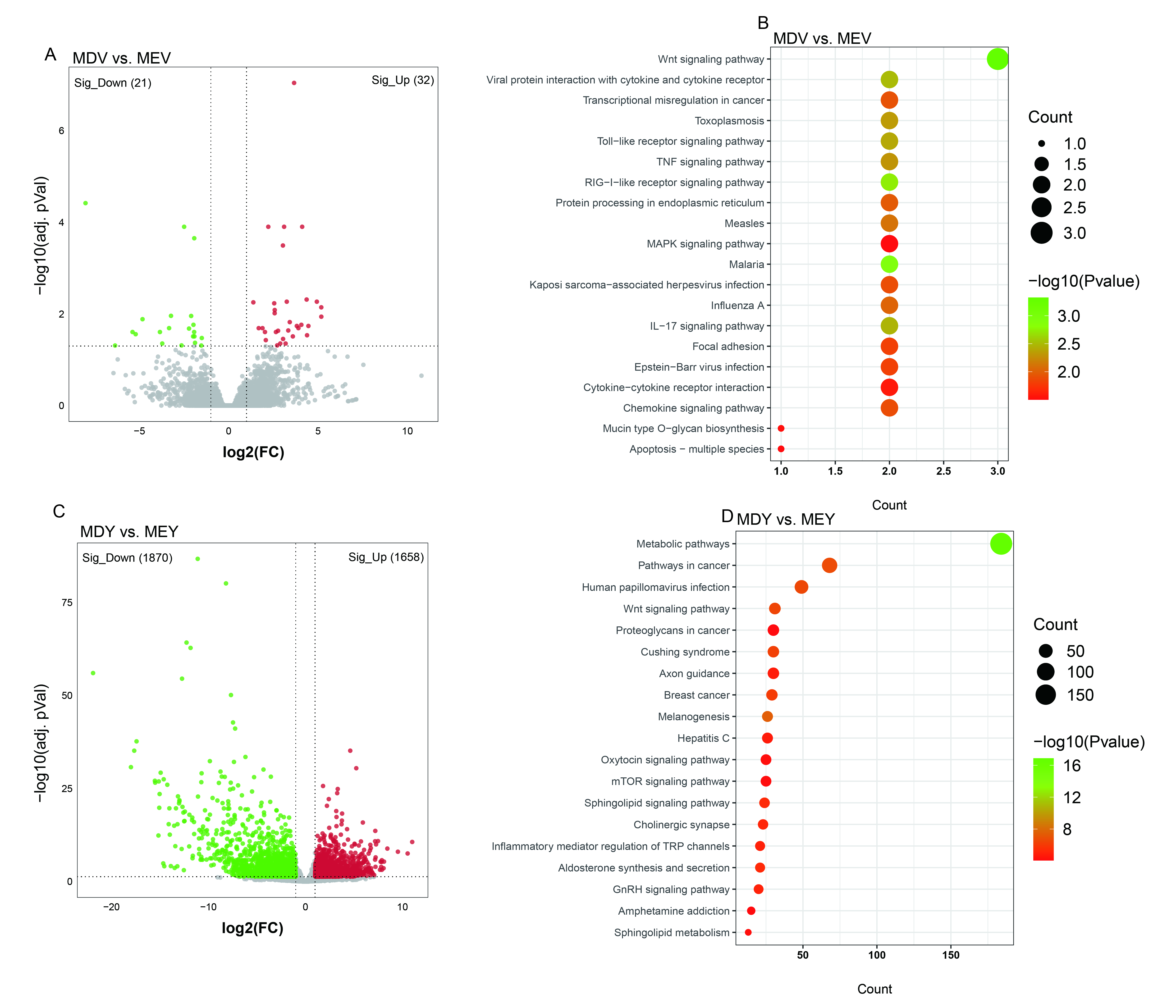

Supplement: Supplementary file 1 [file animals-14-00791-s001.zip › figures1.tif]

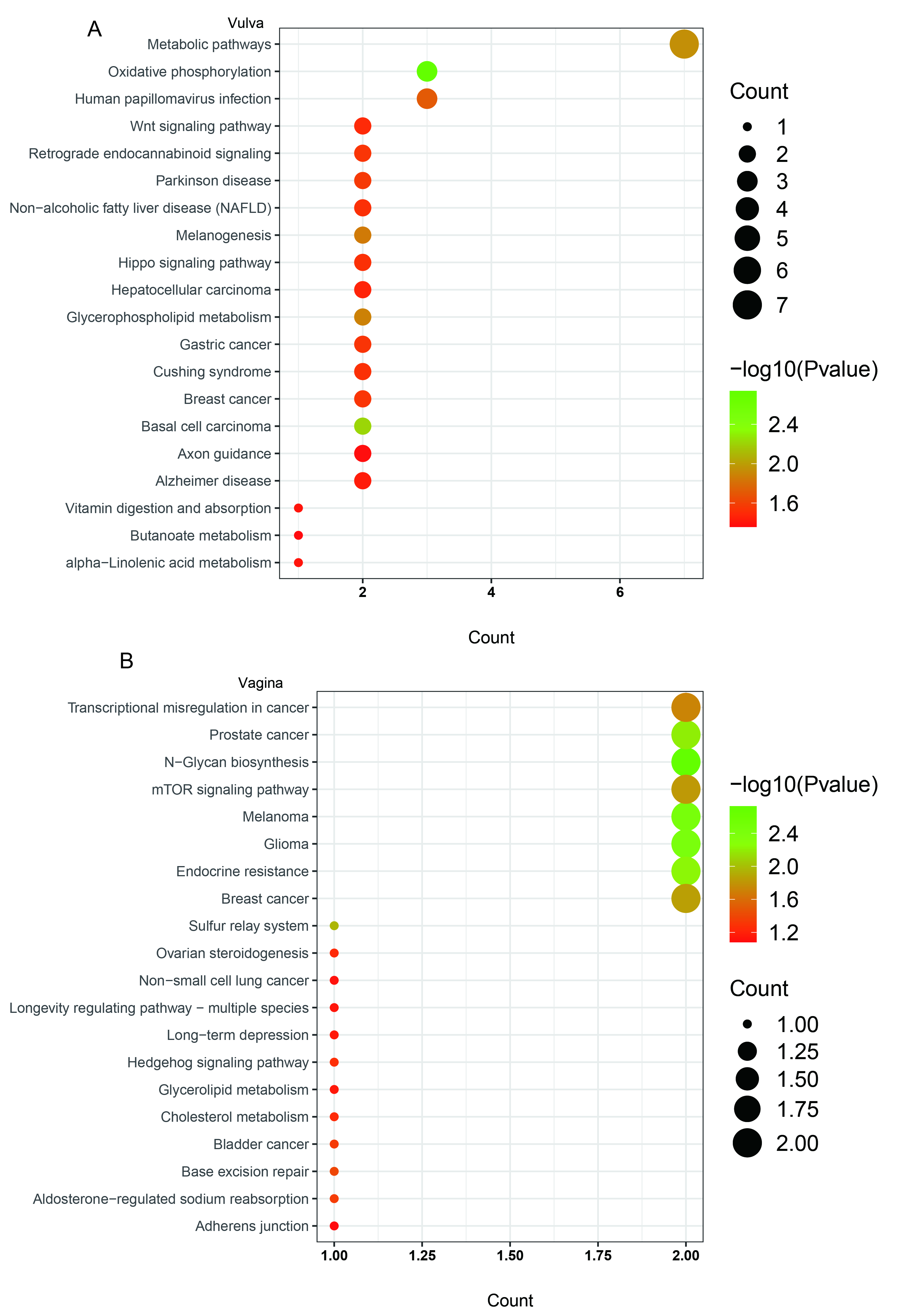

Supplement: Supplementary file 1 [file animals-14-00791-s001.zip › figures2.tif]

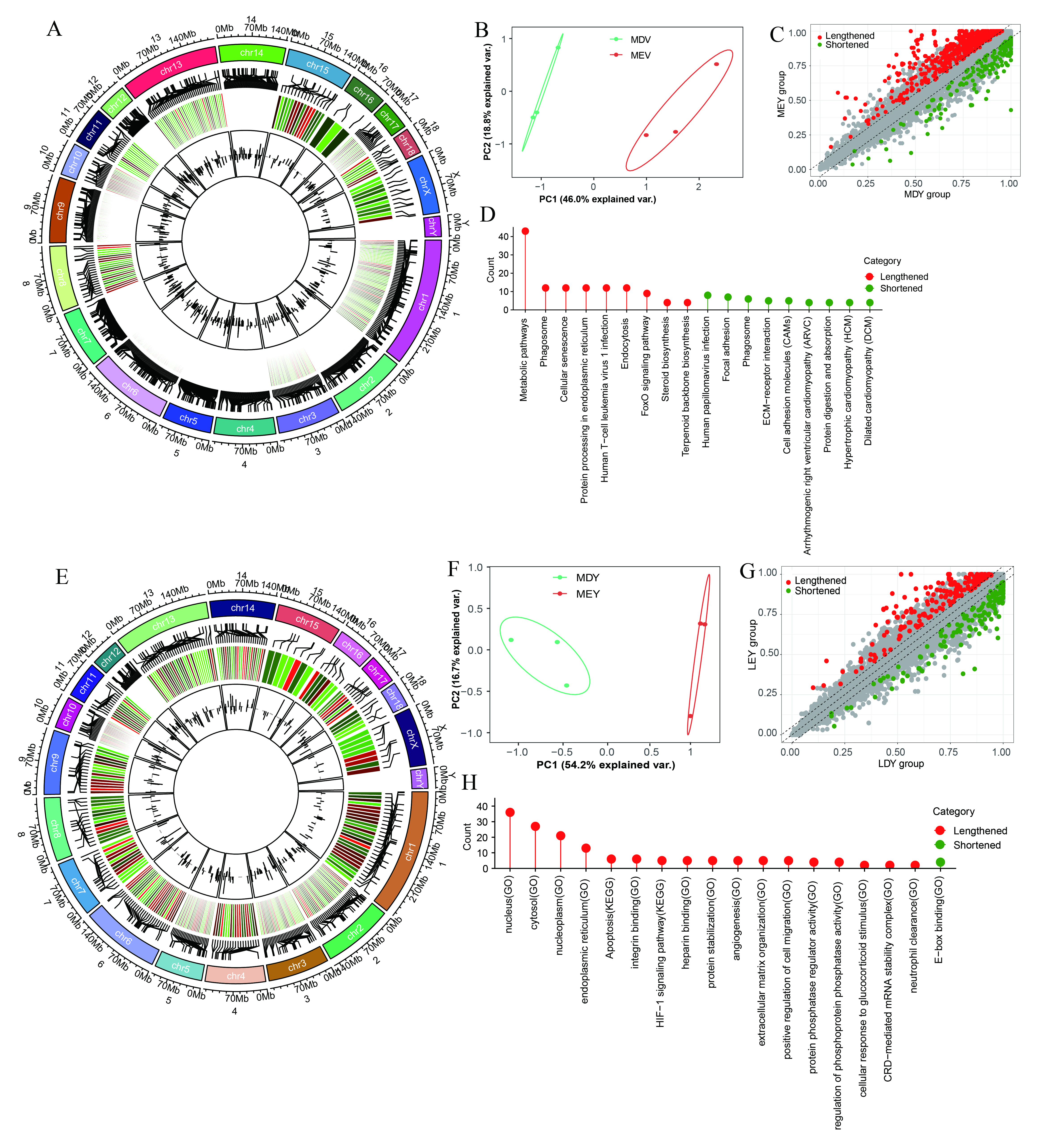

Supplement: Supplementary file 1 [file animals-14-00791-s001.zip › figures3.tif]

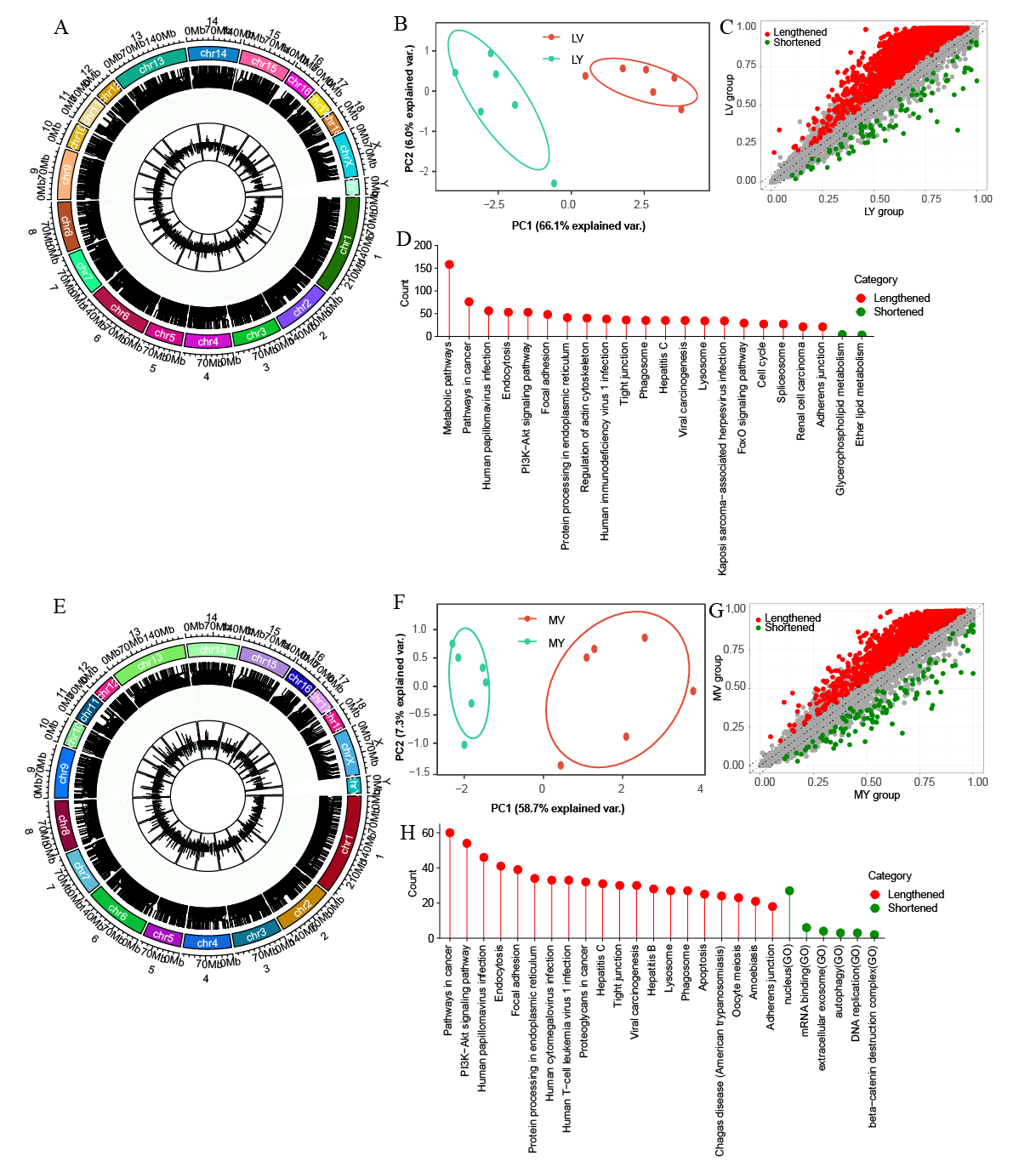

Supplement: Supplementary file 1 [file animals-14-00791-s001.zip › figures4.tif]

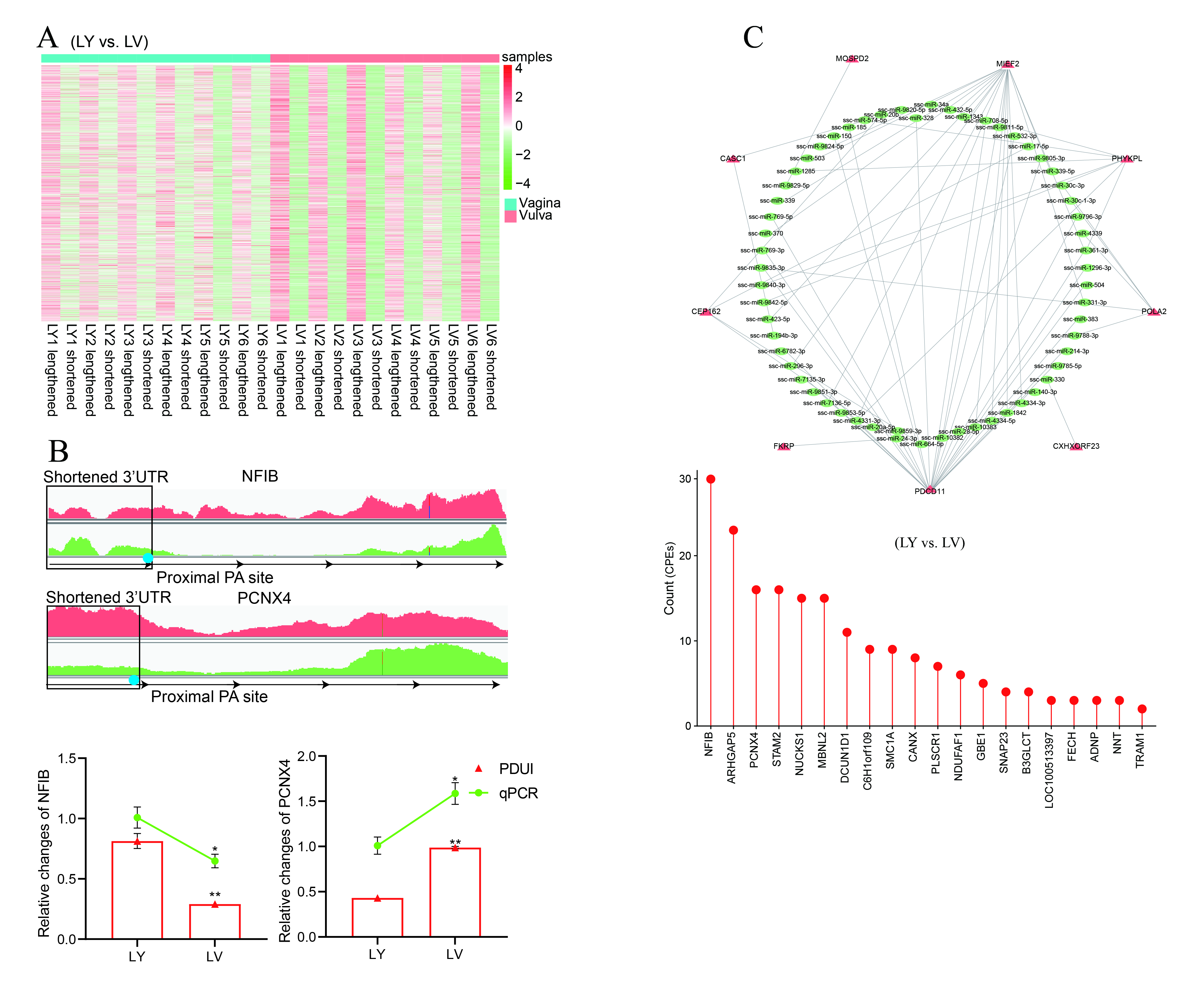

Supplement: Supplementary file 1 [file animals-14-00791-s001.zip › figures5.tif]
